# A novel *SEMA6B* variant causes adult-onset progressive myoclonic epilepsy-11 in a Chinese family: A case report and literature review

**DOI:** 10.3389/fgene.2023.1110310

**Published:** 2023-02-15

**Authors:** Yirao Chen, Xingyan Yang, Xinxiang Yan, Lu Shen, Jifeng Guo, Qian Xu

**Affiliations:** ^1^ Department of Neurology, Xiangya Hospital, Central South University, Changsha, China; ^2^ Department of Neurology, Central Hospital, Bai Yin, China; ^3^ National Clinical Research Center for Geriatric Disorders, Xiangya Hospital, Central South University, Changsha, China; ^4^ Key Laboratory of Hunan Province in Neurodegenerative Disorders, Central South University, Changsha, China; ^5^ Hunan International Scientific and Technological Cooperation Base of Neurodegenerative and Neurogenetic Diseases, Changsha, China; ^6^ Engineering Research Center of Hunan Province in Cognitive Impairment Disorders, Central South University, Changsha, China; ^7^ Key Laboratory of Organ Injury, Aging and Regenerative Medicine of Hunan Province, Changsha, China; ^8^ Centre for Medical Genetics and Hunan Key Laboratory of Medical Genetics, School of Life Sciences, Central South University, Changsha, China

**Keywords:** progressive myoclonic epilepsy, adult onset, SEMA6B, missense variant, case report

## Abstract

This study describes a patient with progressive myoclonic epilepsy-11 (EPM-11), which follows autosomal dominant inheritance caused by a novel *SEMA6B* variant. Most patients develop this disease during infancy or adolescence with action myoclonus, generalized tonic-clonic seizures (GTCS), and progressive neurological deterioration. No cases of adult-onset EPM-11 have been reported yet. Here, we present one case of adult-onset EPM-11 who experienced gait instability, seizures, and cognitive impairment, and harbored a novel missense variant, c.432C>G (p.C144W). Our findings provide a foundation for a better understanding of the phenotypic and genotypic profiles of EPM-11. Further functional studies are recommended to elucidate the pathogenesis of this disease.

## Introduction

Progressive myoclonic epilepsies (PMEs) are a rare group of clinically and genetically heterogeneous disorders characterized by symptoms such as action myoclonus, GTCS, and progressive neurological deterioration (Andermann, 1990), typical onset is in childhood or adolescence. The concept of PMEs was first introduced by Herman Lundborg (Genton et al., 2016), who studied several Swedish families with a common ancestor in 1903 and noticed a particular form of epilepsy associated with progressive myoclonus, with varying degrees of severity. PMEs are typically inherited in an autosomal recessive manner, while a small number of patients have mitochondrial or autosomal dominant inheritance patterns (Franceschetti et al., 2014; Kälviäinen, 2015).

PMEs can be divided into two broad clinical groups. In the first group, the patients present with severe, treatment-resistant, and physically disabling myoclonus, tonic-clonic seizures, and ataxia, with intact cognitive skills (Berkovic et al., 1986). In the second group, the patients experience significant cognitive impairment and degeneration. In the early stages of PMEs, the clinical and electroencephalogram (EEG) characteristics may be similar to those of idiopathic generalized epilepsy syndromes, particularly juvenile myoclonic epilepsy. However, treatment failure, progressive aggravation of neurological symptoms, and EEG manifestations indicate PMEs (Shahwan et al., 2005). As for the management, classical anti-myoclonic agents, including valproate, and levetiracetam, often have limited lasting efficacy in patients with PME. Clonazepam is often helpful, but it typically leads to considerable sedation and tolerance over time. Zonisamide has good anti-epileptic effectiveness, sometimes with a long-lasting effect, and has been shown to improve inter-ictal myoclonus (Vossler et al., 2008; Herzog et al., 2021). Gene modification and enzyme replacement therapies may help improve the condition in the near future (Minassian, 2014).

In recent years, the clinical application of next-generation sequencing technology has led to the discovery of multiple gene mutations related to PME (such as *GOSR2*, *ASAH1*, *KCTD7*, *TBC1D24*, *SCARB2*, *PRICKLE1*, *CARS2*, and *SERPINI*) (Li et al., 2021). Pathogenic variants of semaphorin 6B (*SEMA6B*) can also cause EPM-11 [OMIM#618876], as demonstrated in a few case studies (Hamanaka et al., 2020; Courage et al., 2021; Herzog et al., 2021; Li et al., 2021; Shu et al., 2021; Xiaozhen et al., 2021; Duan et al., 2022). To date, 13 cases of *SEMA6B*-related PME have been reported, all of whom presented with seizures, while three presented with myoclonic seizures. Adult-onset EPM-11 has rarely been reported in the previous literature, and herein, we report one case of adult-onset EPM-11 who presented with late-onset gait instability, seizures, and cognitive impairment.

### Case description

**FIGURE 1:**
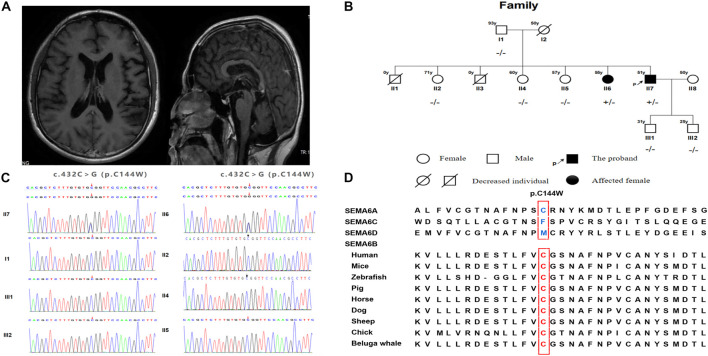
**(A)** Brain MRI of the proband. **(B)** SEMA6B gene sequencing of the family members. **(C)** Genetic pedigree of the family and corresponding individual genotypes. **(D)** The alignment of SEMA6B amino acid sequences.

A 51-year-old Chinese man who presented with gait disturbance and GTCS was admitted to our neurology department. He first presented with clinical symptoms when he was 46 years old and experienced difficulty in walking steadily. The symptom slightly relieved after treatment with vitamins B1 and B12. His personality was dampened, and his memory deteriorated over time. He presented with generalized seizures at 50 years of age, which occurred twice a year. Physical examination revealed horizontal nystagmus, positive palmomental reflexes, postural and intention tremors, increased muscle tension in both lower extremities, tendon hyperreflexia, positive ankle clonus and heel-knee-shin tests of the right side, and dysmetria in the finger-to-nose test. Somatosensory evoked potential (SEP) showed giant evoked potentials in the bilateral upper limbs and enhanced C reflexes after stimulation of the median nerve bilaterally. A 24 h EEG showed bilaterally sharp waves over the fronto-centro-parietal electrodes, particularly over the right regions. Magnetic resonance imaging (MRI) revealed mild cortical and cerebellar atrophy (Figure 1A). The cognitive function was assessed using the Mini-Mental State Examination, with a score of 27, and the Montreal Cognitive Assessment, with a score of 23. We commenced treatment with levetiracetam (500 mg/d) and B vitamins. The patient had no seizures in 9 months, and his symptoms did not aggravate with follow-up.

There were no reports of consanguineous marriages within the patient’s family. Before the patient’s birth, his mother had two miscarriages. One of the proband’s sisters began having seizures, gait disturbances, and cognitive impairment in her 50s (Figure 1B). She experienced generalized seizures more frequently and had more severe cognitive impairment than the proband. She underwent antiepileptic treatment without any symptomatic improvement. Her condition worsened over time, and she had been bedridden for 2 years. Due to her cognitive function decline, she was unable to cooperate with us to complete the physical examination. However, her muscle tension was increased in all limbs, and the pathological signs were positive. The remaining members of the proband’s family were unaffected, while his mother died at 50 years of age in an accident. After obtaining consent, we performed further genetic analysis. We completed cytosine-adenine-guanine (CAG) repeat expansion detection and found no spinocerebellar ataxia (SCA)-related dynamic mutations; in contrast, whole exome sequencing (WES) detected a missense variant, c.432C>G (p.C144W), in exon six of *SEMA6B* in the proband. The proband’s sister carried the same heterozygous mutation (Figure 1C).

## Discussion

PME has high genetic heterogeneity, and more than 40 genes are reportedly associated with this disorder. *SEMA6B* is one of the pathogenic genes that are related to PME and is located on chromosome 19p13; it contains 17 coding exons and a PPAR-binding site in the upstream sequence (Correa et al., 2001). *SEMA6B* is a member of the class-6 semaphorin family, which is involved in neural development, including neural crest cell migration, axon guidance, and cerebellar development (Andermatt et al., 2014). *SEMA6B* is highly expressed in multiple brain regions, including the cerebral cortex, cerebellar Purkinje cells, interneurons, and specific cell types, including excitatory and GABAergic inhibitory neurons (Hamanaka et al., 2020). Consequently, disruption of *SEMA6B* function in GABAergic neurons may contribute to epilepsy.

To the best of our knowledge, 13 cases of *SEMA6B*-associated PME, including the case described in the present study, have been reported. The first 12 had childhood-to-juvenile onset, and only our case presented as adult onset. The clinical characteristics and genetic detection of the EMP-11 patients are summarized in Table 1. Several reported cases identified patients harboring a truncating variant in the final exon of *SEMA6B*. Interestingly, a missense variant in exon 16 of *SEMA6B* (c.1834G > A/p. V612M) was found to be related to cerebellar hypoplasia, with symptoms including cerebellar ataxia and developmental delay (Aldinger et al., 2019). The *SEMA6B* c.432C>G (exon6) variant identified in this study is a novel mutation and is absent in the Genome Aggregation Database (gnomAD), Exome Aggregation Consortium (ExAC), and 1000 Genomes Project. This variant is predicted to be a damaging missense mutation by several missense prediction software packages, including Polythen2, MutationTaster, CADD, and ReVe, and p. C144W is highly conserved in other organisms (Figure 1D).

**TABLE 1:** Clinical features of the patient reported in this work and comparison with published cases of SEMA6B-related progressive myoclonic epilepsy.

Patient number	Nationality	Sex	Pathogenic variant	Inheritance	Age of onset	Initial symptom		Regression	Seizure types	Ataxia	Intention tremor	Rigidity	Spasticity	Increased deep tendon reflex	Pathogenic reflex	Brain MRI	EEG	SEP
1 (12)	Japanese	Male	c.1950-1969dup (p.Arg657Profs*35)	*De novo*	6 years	Seizure and developmental delay		Y (motor skill and dysarthria)	GTCS, absence seizures, and atonic seizures	Y	Y	Y	Y	Y (upper and lower limbs)	Y (Rossolimo sign:positive,mendel-bekhterev sign:positive)	Normal	Abnormal	Prolonged N20 latency and high amplitude of P24-N33
2 (12)	Japanese	Female	c.1976-1982del (p.Ala659-Valfs*24)	*De novo*	11 months	Seizure		Y (motor skill)	GTCS, loss of consciousness with abnormal eye movement, and complex partial seizure and atonic seizure	Y	Y	Y	Y	Y (upper and lower limbs)	N	Mild cerebellar atrophy	Abnormal	Giant SEP
3 (12)	Israeli	Male	c.1991del (p.Gly664Alafs*21)	*De novo*	2 years	Seizure		Y (motor and verbal skill)	Absence seizures	Y	Y	NP	NP	NP	NP	Small vermis	Abnormal	NP
4 (12)	Malaysian	Female	c.1991del (p.Gly664 Alafs*21)	*De novo*	4 years	Seizure and developmental delay		Y	Atonic seizure	Y	Y	NP	NP	N	N	Normal	Abnormal	NP
5 (10)	Chinese	Female	c.1960 1978del (p.Leu654Argfs*25)	*De novo*	4 years	Seizure and developmental delay		Y	Atonic seizures	Y	Y	N	N	N	N	Herniation of the cerebellar tonsils	Abnormal	U
6 (8)	German	Female	c.2067G>A (p. Trp689Ter)	U	10 years	Seizure and developmental delay		Y (motor and verbal skill)	Nonconvulsive/dyscognitive status epilepticus and myoclonus	Y	U	Y	U	U	U	Normal	Abnormal	NP
7 (11)	Chinese	Female	c.2056C>T (p.Gln686∗)	*De novo*	2 years	Seizure and developmental delay		Y (motor and intellectual)	Atonic seizure, complex partial seizure, and atypical absence seizure	N	U	U	U	U	U	Normal	Abnormal	NP
8 (11)	Chinese	Male	c.1483G>T (p.Gly495Trp)	*De novo*	2 years	Seizure		N	Complex partial seizure	N	N	N	N	N	N	Normal	NP	NP
9 (13)	Australia	Male	c.1993del (p.Arg665GLyfs*20)	*De novo*	2.5 years	Seizure and developmental delay		Y	Drop attacks and absence seizures, and multifocal myoclonus	Y	N	U	U	U	U	N	N	N
10 (13)	Canada	Female	c.2032del (p.Glu678Argfs*7)	U	2.5 years	Seizure and developmental delay		N	Drop attacks and absence seizures, and multifocal myoclonus	Y	U	U	U	U	U	N	N	N
11 (14)	Chinese	Male	c.1934del (p.G645fs)	*De novo*	6 months	Seizure and developmental delay		Y (motor and verbal skill)	GTCS and febrile seizure	U	U	U	U	U	U	Normal	Abnormal	N
12 (15)	Chinese	Male	c.2023delG (p.V675fs)	*De novo*	3 years	Seizure and developmental delay		Y (motor and verbal skill)	Focal seizures, atonic seizures, atypical absence seizures, and nonconvulsive status epilepticus	Y	Y	U	Y	U	U	Normal	Abnormal	N
13	Chinese	Male	c.432 C>G (p.Cys144Trp)	U	46 years	Seizure		N	GTCS	Y	Y	Y	N	Y	Y	Brain atrophy; blurring bilateral substantia nigra swallow sign; relatively sparing of nerve fibers in the right pyramidal tract	Abnormal	Giant evoked potentials in bilateral upper limbs and C waves after stimulating the bilateral median nerve

EEG electroencephalogram, GTCS generalized tonic-clonic seizures, MRI magnetic resonance imaging, SEP somatosensory evoked potential, NP not performed, U unknown, Y present, N not present.

In this family, the proband and his sister have similar phenotypes and carry the same variant, which is absent in other siblings. Therefore, we assume that this variant might have originated from their mother, who died in an accident. The mechanism of SEMA6B-related disease remains unclear. Previous studies have shown that missense and nonsense variants can lead to protein function problems, resulting in clinical symptoms such as epilepsy (Xiaozhen et al., 2021). Further functional studies will help us clarify the mechanism.

## Conclusion

In conclusion, we reported a family with adult-onset PME-11 harboring a novel heterozygous missense variant. The new genetic variation reported here strengthens the gene–disease relationship. This finding may expand the consideration of the age of onset for EPM-11 and extend the mutational spectrum. However, further functional studies are required to better elucidate the pathogenesis of this disease.

## Data Availability

The original contributions presented in the study are included in the article/Supplementary Materials, further inquiries can be directed to the corresponding author.

## References

[B1] Aldinger K. A. Timms A. E. Thomson Z. Mirzaa G. M. Bennett J. T. Rosenberg A. B. (2019). Redefining the etiologic landscape of cerebellar malformations. Am. J. Hum. Genet. 105 (3), 606–615. 10.1016/j.ajhg.2019.07.019 31474318 PMC6731369

[B2] Andermatt I. Wilson N. H. Bergmann T. Mauti O. Gesemann M. Sockanathan S. (2014). Semaphorin 6B acts as a receptor in post-crossing commissural axon guidance. Dev. Camb. Engl. 141 (19), 3709–3720. 10.1242/dev.112185 PMC6514406 25209245

[B3] Berkovic S. F. Andermann F. Carpenter S. Wolfe L. S. (1986). Progressive myoclonus epilepsies: Specific causes and diagnosis. N. Engl. J. Med. 315 (5), 296–305. 10.1056/NEJM198607313150506 3088452

[B4] Andermann (1990), Classification of progressive myoclonus epilepsies and related disorders. Marseille Consensus Group. Ann. neurology 28, 113, 10.1002/ana.410280129 2115761

[B5] Correa R. G. Sasahara R. M. Bengtson M. H. Katayama M. L. Salim A. C. Brentani M. M. (2001). Human semaphorin 6B [(HSA)SEMA6B], a novel human class 6 semaphorin gene: Alternative splicing and all-trans-retinoic acid-dependent downregulation in glioblastoma cell lines. Genomics 73 (3), 343–348. 10.1006/geno.2001.6525 11350127

[B6] Courage C. Oliver K. L. Park E. J. Cameron J. M. Grabińska K. A. Muona M. (2021). Progressive myoclonus epilepsies-Residual unsolved cases have marked genetic heterogeneity including dolichol-dependent protein glycosylation pathway genes. Am. J. Hum. Genet. 108 (4), 722–738. 10.1016/j.ajhg.2021.03.013 33798445 PMC8059372

[B7] Duan J. Chen Y. Hu Z. Ye Y. Zhang T. Li C. (2022). Non-convulsive status epilepticus in SEMA6B-related progressive myoclonic epilepsy: A case report with literature review. Front. Pediatr. 10, 859183. 10.3389/fped.2022.859183 35573939 PMC9096209

[B8] Franceschetti S. Michelucci R. Canafoglia L. Striano P. Gambardella A. Magaudda A. (2014). Progressive myoclonic epilepsies: Definitive and still undetermined causes. Neurology 82 (5), 405–411. 10.1212/WNL.0000000000000077 24384641 PMC3917687

[B9] Genton P. Striano P. Minassian B. A. (2016). The history of progressive myoclonus epilepsies. Epileptic Disord. Int. epilepsy J. videotape 18 (S2), 3–10. 10.1684/epd.2016.0834 PMC5777179 27621064

[B10] Hamanaka K. Imagawa E. Koshimizu E. Miyatake S. Tohyama J. Yamagata T. (2020). De novo truncating variants in the last exon of SEMA6B cause progressive myoclonic epilepsy. Am. J. Hum. Genet. 106 (4), 549–558. 10.1016/j.ajhg.2020.02.011 32169168 PMC7118575

[B11] Herzog R. Hellenbroich Y. Brüggemann N. Lohmann K. Grimmel M. Haack T. B. (2021). Zonisamide‐responsive myoclonus in SEMA6B‐associated progressive myoclonic epilepsy. Ann. Clin. Transl. Neurology 8 (7), 1524–1527. 10.1002/acn3.51403 PMC8283161 34092044

[B12] Kälviäinen R. (2015). Progressive myoclonus epilepsies. Seminars Neurology 35 (03), 293–299. 10.1055/s-0035-1552620 26060909

[B13] Li Q. Liu M. Huang D-P. Li T. Huang J. Jiang P. (2021). A de novo SEMA6B variant in a Chinese patient with progressive myoclonic epilepsy-11 and review of the literature. J. Mol. Neurosci. 71 (9), 1944–1950. 10.1007/s12031-021-01880-0 34218423

[B14] Minassian B. A. (2014). The progressive myoclonus epilepsies. Prog. Brain Res. 213, 113–122. 10.1016/B978-0-444-63326-2.00006-5 25194486

[B15] Shahwan A. Farrell M. Delanty N. (2005). Progressive myoclonic epilepsies: A review of genetic and therapeutic aspects. Lancet Neurology 4 (4), 239–248. 10.1016/S1474-4422(05)70043-0 15778103

[B16] Shu L. Xu Y. Tian Q. Chen Y. Wang Y. Xi H. (2021). A frameshift variant in the SEMA6B gene causes global developmental delay and febrile seizures. Neurosci. Bull. 37 (9), 1357–1360. 10.1007/s12264-021-00717-5 34110594 PMC8423932

[B17] Vossler D. G. Conry J. A. Murphy J. V. ZNS-502/505 PME Study Group (2008). Zonisamide for the treatment of myoclonic seizures in progressive myoclonic epilepsy: An open-label study. Epileptic Disord. Int. epilepsy J. videotape 10 (1), 31–34. 10.1684/epd.2008.0168 18367429

[B18] Xiaozhen S. Fan Y. Fang Y. Xiaoping L. Jia J. Wuhen X. (2021). Novel truncating and missense variants in SEMA6B in patients with early-onset epilepsy. Front. Cell Dev. Biol. 9, 633819. 10.3389/fcell.2021.633819 34017830 PMC8129541

